# *Moniezia benedeni* Infection Restrain IgA^+^, IgG^+^, and IgM^+^ Cells Residence in Sheep (*Ovis aries*) Small Intestine

**DOI:** 10.3389/fvets.2022.878467

**Published:** 2022-04-28

**Authors:** Luo-Xia Han, Wan-Ling Yao, Jing Pan, Bao-Shan Wang, Wan-Hong He, Xi-Ping Fan, Wen-Hui Wang, Wang-Dong Zhang

**Affiliations:** College of Veterinary Medicine, Gansu Agricultural University, Lanzhou, China

**Keywords:** parasitology, worms, *Moniezia benedeni*, sheep small intestine, IgA^+^ cell, IgG^+^ cell, IgM^+^ cell, immunosuppression

## Abstract

Secreted immunoglobulin A (SIgA), IgG, and IgM play a crucial role in forming the intestinal mucosal immune barrier, and parasites could disturb the host's immune response by releasing various immunomodulatory molecules. *Moniezia benedeni* is an important pathogen parasitizing in the sheep small intestine. It is aimed to explore the residence characteristics of IgA^+^, IgG^+^, and IgM^+^ cells in the sheep small intestine, and the influence of *Moniezia benedeni* infection on them. Control group (*n* = 6) and infected group (*n* = 6) were selected, respectively, and the three subtype cells residing in the small intestine were systematically observed and analyzed. The results showed that in the Control group, the three types of positive cells were all distributed diffusely, and the total densities in jejunum, duodenum and ileum was gradually declined in turn. Notably, the change trend of IgA^+^ and IgG^+^ cells densities were both congruent with the total densities, and the differences among them were significant, respectively (*P* < 0.05); the IgM^+^ cells density was the highest in duodenum, followed by jejunum and ileum, there was no significant difference between duodenum and jejunum (*P* > 0.05), but both significantly higher than in ileum (*P* < 0.05). In the Infected group, their total densities in duodenum, jejunum and ileum were gradually declined in turn. Notably, the IgA^+^ and IgM^+^ cells densities change trend was the same as the total densities, and the differences among them were significant, respectively (*P* < 0.05). The IgG^+^ cells density in duodenum was the highest, followed by ileum and jejunum and there was significantly difference among them (*P* < 0.05). The comparison results between Control and Infected groups showed that from the duodenum, jejunum to ileum, IgA^+^, IgG^+^, and IgM^+^ cells were all reduced significantly, respectively. The results suggest that the three types of positive cells were resided heterogeneously in the small intestinal mucosa, that is, significant region-specificity; *Moniezia benedeni* infection could not change their diffuse distribution characteristics, but strikingly, reduce their resident densities, and the forming mucosal immune barrier were significantly inhibited. It provided powerful evidence for studying on the molecular mechanism of *Moniezia benedeni* evasion from immune surveillance by strongly inhibiting the host's mucosal immune barrier.

## Introduction

In humans and animals, the gut is an important organ for digesting food and absorbing nutrients. However, due to its direct communication with the external environment, it is always faced with the threat of a large number of potential antigens, such as food antigens (1, 2), toxins (3, 4), bacteria (5–7), viruses (8, 9), fungi (10–13), and parasites and their metabolites (14–16). The gastrointestinal mucosal immune system, could provide comprehensive and effective protection for gastrointestinal tract through dynamically regulating the immune homeostasis and forming multi-layer defense barriers, such as mechanical and physical barriers (17, 18), chemical barriers (19) and immune barrier (20), etc. *via* the precise and sensitive identification and monitoring. Importantly, a variety of secreted antibodies, mainly including secreted immunoglobulin A (SIgA) (21–25), IgG (26, 27), and IgM (28, 29), were mediated and produced by the adaptive immune response and transported to the gut luminal surface by their specific transport receptors such as poly immunoglobulin receptor (pIgR) (30–32) and neonatal Fc receptor (FcRn) (33). They could regulate intestinal homeostasis and play an immune defense barrier through neutralizing and regulating the composition and density of intestinal commensal bacteria, inhibiting the inflammatory response, and preventing the commensal bacteria and pathogenic bacteria from entering the mucosa. Studies have shown that if they were abnormally secreted, a variety of diseases could be induced, for instance, ileal flora disorder (34) and inflammation (35) was caused by IgA deficiency; the concentration of SIgM in mice feces was related to flora imbalance and autoimmune diseases (29).

However, emerging researches reported that there is a characteristic of segmental immune defense in different intestinal regions. Such as mesenteric lymph nodes (36, 37), isolated lymphoid follicles, and dendritic cells (38), macrophages (39), Th17 and Treg (40), distributed in *laminar propria* (LP) of different intestinal segments, all exhibited regional specificity and induced different immune responses. Moreover, the SIgA, IgG, and IgM forming the immune defense barrier differed in different intestinal segments (41, 42). For instance, our previous studies showed that in Bactrian camel intestine the distribution density of IgA^+^ cells was the highest and predominant, while IgG^+^ cells was relatively low, and their densities were also not uniform in the duodenum, jejunum, and ileum (43). Therefore, the intestinal mucosal immunity presented obvious regional specificity in multiple aspects such as immune induction, response, and effect in different intestinal segments.

Numerous studies have shown that helminths could release a variety of immunoregulatory factors, which could affect the initiation of the host's anti-parasite immune responses and affect antigen recognition and processing, and adaptive immune responses (14). For instance, it has been shown that succinate produced by parasite could activate the tuft cell-group 2 innate lymphoid cell (ILC2) loop and induce a type 2 immune response in the host gut (44–46). The adult *Moniezia benedeni*, one of the most important pathogens of tapeworm of sheep, parasitizing in the small intestine of sheep, consists of three parts: the scolex, neck and strobila. It mostly infects lambs aged 5 to 7 months, and can cause death in severe cases. The disease is distributed all over the world and is mostly endemic. In the present study, the distribution of IgA^+^, IgG^+^, and IgM^+^ cells in the small intestine of sheep, and their changes induced by *Moniezia benedeni* infection were observed and analyzed. These data would provide the necessary support for further revealing the impact of *Moniezia benedeni* infection on the intestinal immune defense barrier of sheep, and lay the groundwork for further researching the regulatory mechanism of the intestinal mucosal immune system of sheep in response to *Moniezia benedeni* infection.

## Materials and Methods

### Reagents and Chemicals

#### Primary Antibody

(1) Rabbit Anti-Sheep IgM (Ab112760, Lot No. GR3252337-3, abcam, USA), the best working concentration was determined to be 1:1500.
(2) Rabbit anti-sheep IgA polyclonal antibody (AB112756, Lot No. GR3302257-1, abcam, USA), the best working concentration was determined to be 1:2000.
(3) Rabbit Anti-Shee IgG (BA1040, Lot No. BST13A25C15K40, Boster, Wuhan, Hubei, China), the best working concentration was determined to be 1:200.

#### Secondary Antibody

SABC-POD (mouse/rabbit IgG) kit (SA1020, Lot No. 16C10F19B0120, Boster, Wuhan, Hubei, China). The kit contains: (1) 3% H_2_O_2_ 12 ml, for removing endogenous peroxidase; (2) 5% BSA blocking solution: 12 ml, for blocking tissue sections; (3) biotinylated goat anti-mouse/rabbit IgG polyclonal antibody: 12 ml; (4) SABC: 12 ml. The reagent is a ready-to-use kit, and the secondary antibody is used directly without dilution.

### Experimental Design and Sample Preparation

According to strobila in feces, the naturally infected sheep (8–10 months old, female) were collected in July to September, which is the highest incidence period of the *Moniezia* infection in Wuwei, Gansu province. The selected sheep were anesthetized intravenously with sodium pentobarbital (20 mg/kg) and then exsanguinated in carotid artery to death. The abdomen of each sheep was cut open, and the whole small intestine were obtained. The intestinal contents were removed and the mucosal surface was thoroughly washed by physiological saline. The *Moniezia* were collected and fixed in 75% ethanol for species identification; all histological samples of the duodenum, jejunum and ileum were fixed in a 4% neutral paraformaldehyde solution. According to the results of the species identification of the tapeworm, the infected group (*n* = 6) infected with *Moniezia benedeni* (tapeworm number in each sheep <3) were finally selected, and the co-infection samples were excluded (such as *Helictometra*). The identification of *Moniezia benedeni* was based on the intersegmental glands following hematoxylin staining (47, 48). Meanwhile, the control group (*n* = 6) was also collected from the healthy sheep in the same flock (8–10 months old, female). The methods of obtaining histological samples were the same as infected group. And the paraffin sections were made by conventional methods.

### Immunohistochemical Staining

The prepared paraffin sections were performed to SABC immunohistochemical staining as follows: for detailed steps, see reference (49). After deparaffination, the sections were treated with 3% H_2_O_2_ for 10 min, washed with distilled water for 3 times; treated with 1 mg/ml 1:250 pancreatin (250 NFU/mg, Sigma, USA) for 30 min at 37 °C, washed with distilled water for 3 times; to avoid non-specific binding of tissue, 5% BSA (from a ready-to-use immunohistochemical staining kit) was added on the tissue samples for blocking at 37 °C for 30 min. The excess fluid was shaken off, and the primary antibodies were added on the sections. After being incubated overnight at 4 °C, the sections were washed with PBS (0.01 mol/L, pH 7.2) for 2 min × 3 times; the secondary antibody (HRP-linked goat anti-mouse/rabbit IgG). After being incubate at 37 °C for 30 min, the sections were washed with PBS for 5 min × 4 times; SABC was added dropwise. After being incubate at 37 °C for 30 min, and the sections were washed with PBS for 5 min × 4 times; colored with DAB kit (20 ×, product number: ZLI-9018, ZSGB-BIO, Beijing, China) at room temperature, after that, it was lightly counterstained with Hematoxylin, then dehydrated, and mounted with neutral balsam.

### Light Microscopy

In each group, the location, and characteristics of the residence of IgA^+^, IgG^+^, and IgM^+^ cells were observed carefully with an Olympus microscope (Olympus, Hamburg, Germany), respectively. Thirty slices of each part (including duodenum, jejunum, and ileum) of each sample in the Normal and Infected groups were observed, and then the photographs were collected.

### Statistical Analysis

Five slices were randomly selected from each site. Ten fields of the microscope were randomly selected for each section and photographed with an Olympus DP-71 photomicrography system. The numbers of IgA^+^, IgG^+^, and IgM^+^ cells in each field were counted, and the respective densities were calculated (Image-Pro Plus 6.0) (49, 50). SPSS 23.0 (SPSS Inc., Chicago, USA) was used for statistics. One-way analysis of variance with Duncan's new multiple range test was used to analyze the density differences of each positive cell among various sites in the same group; independent *T*-test was used to analyze the differences between the Infected group and Normal group for the same site. *P* < 0.05 means significant difference, *P* > 0.05 means no significant difference.

## Results

### Identification of *Moniezia benedeni*

**Figure 1:**
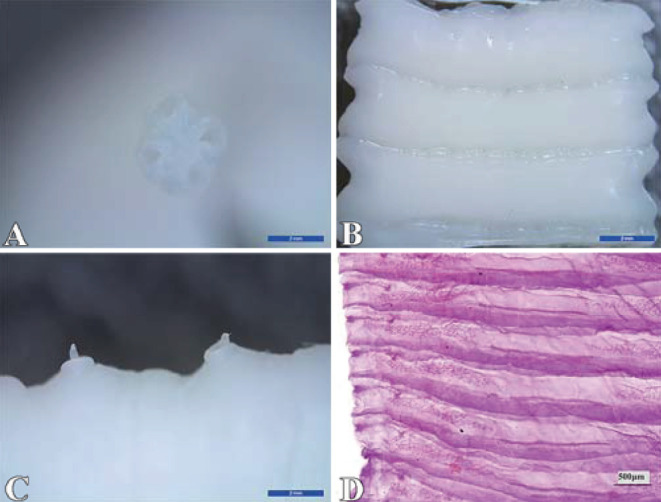
Identification of *Moniezia benedeni*. **(A)** scolex; **(B)** strobila; **(C)** germ antrum; **(D)** interproglottidal glands. The scale bar of (A), (B) and (C) is 2 mm, and (D) is 500 μm.

The results showed that 1 or 2 flatworms parasitizing in the sheep small intestine were milky white or creamy yellow, which has four suckers, no rostellum and hooks, the scolex is small, measuring up to 1 mm in diameter (Figure 1A); segments are broader than they are long (Figure 1B), and contain two sets of genital organs along the lateral margin of each segment (Figure 1C); the inter-proglottid glands are confined to a short row close to the middle of the posterior margin of the segment (Figure 1D), and were identified as *Moniezia benedeni*.

### Distribution Characteristics of IgA^+^, IgG^+^, IgM^+^ Cells in the Small Intestine of Healthy Sheep

**Figure 2:**
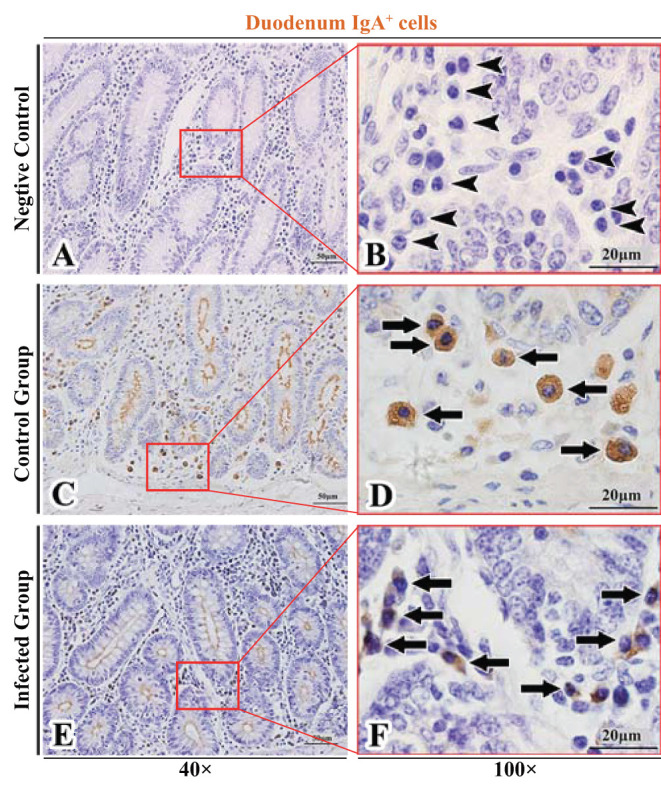
Distribution of IgA^+^ cells in sheep duodenum. **(A,B)** are negative controls; **(C,D)** are Control groups; **(E,F)** are Infected groups. Triangular arrows indicated plasma cells, arrows indicated IgA^+^ cells, and the right image (Scale bar, 20 μm) is a magnification from the part of the left image (Scale bar, 50 μm).

**Figure 3:**
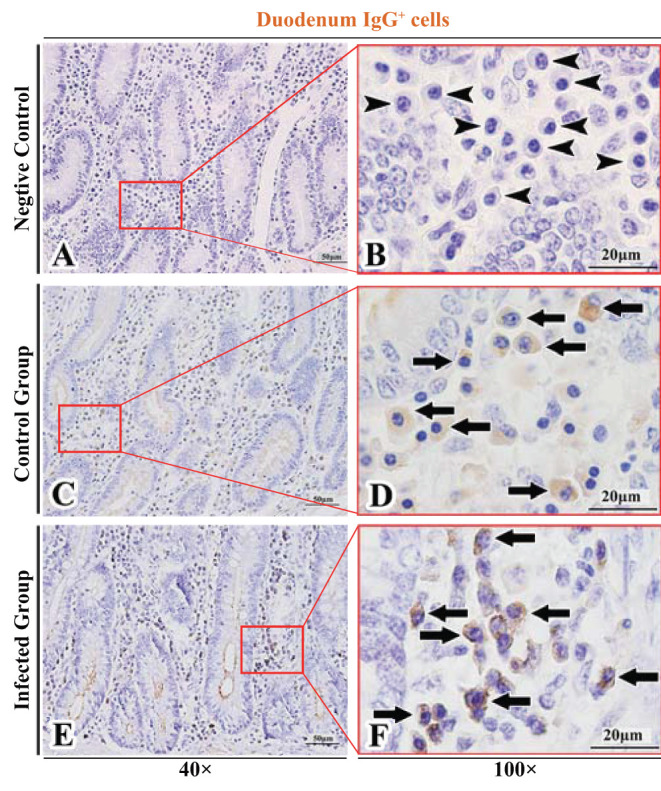
Distribution of IgG^+^ cells in sheep duodenum. **(A,B)** are negative controls; **(C,D)** are Control groups; **(E,F)** are Infected groups. Triangular arrows indicated plasma cells, arrows indicated IgG^+^ cells, and the right image (Scale bar, 20 μm) is a magnification from the part of the left image (Scale bar, 50 μm).

**Figure 4:**
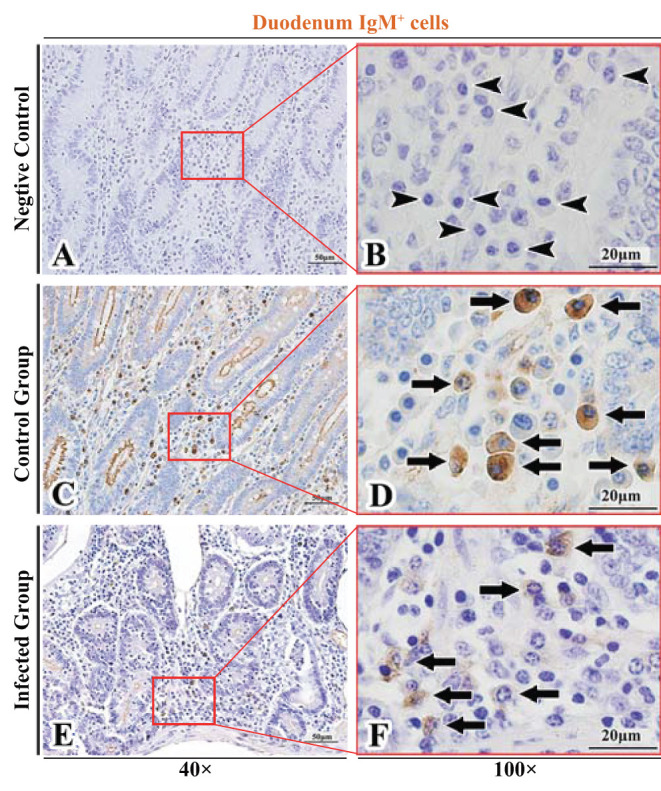
Distribution of IgM^+^ cells in sheep duodenum. **(A,B)** are negative controls; **(C,D)** are Control groups; **(E,F)** are Infected groups. Triangular arrows indicated plasma cells, arrows indicated IgM^+^ cells, and the right image (Scale bar, 20 μm) is a magnification from the part of the left image (Scale bar, 50 μm).

**Figure 5:**
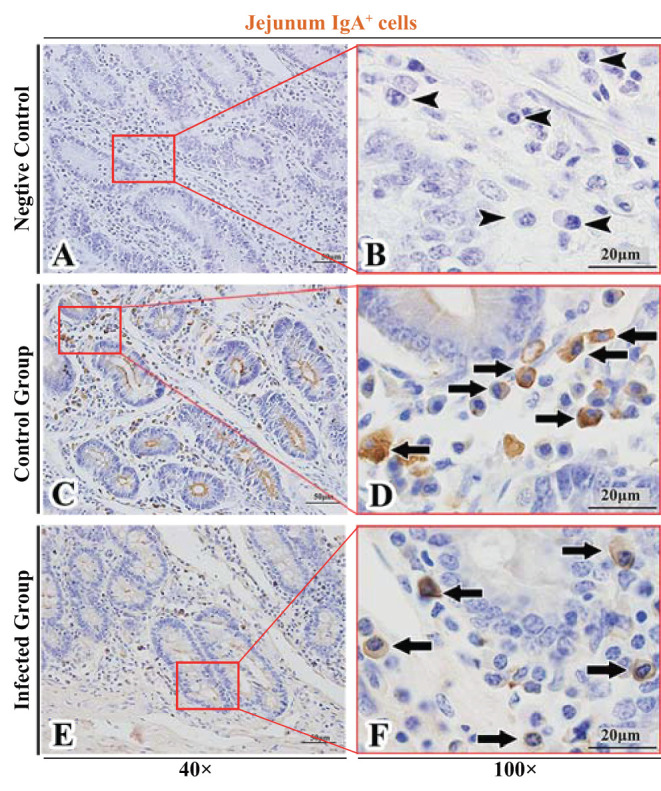
Distribution of IgA^+^ cells in sheep jejunum. **(A,B)** are negative controls; **(C,D)** are Control groups; **(E,F)** are Infected groups. Triangular arrows indicated plasma cells, arrows indicated IgA^+^ cells, and the right image (Scale bar, 20 μm) is a magnification from the part of the left image (Scale bar, 50 μm).

**Figure 6:**
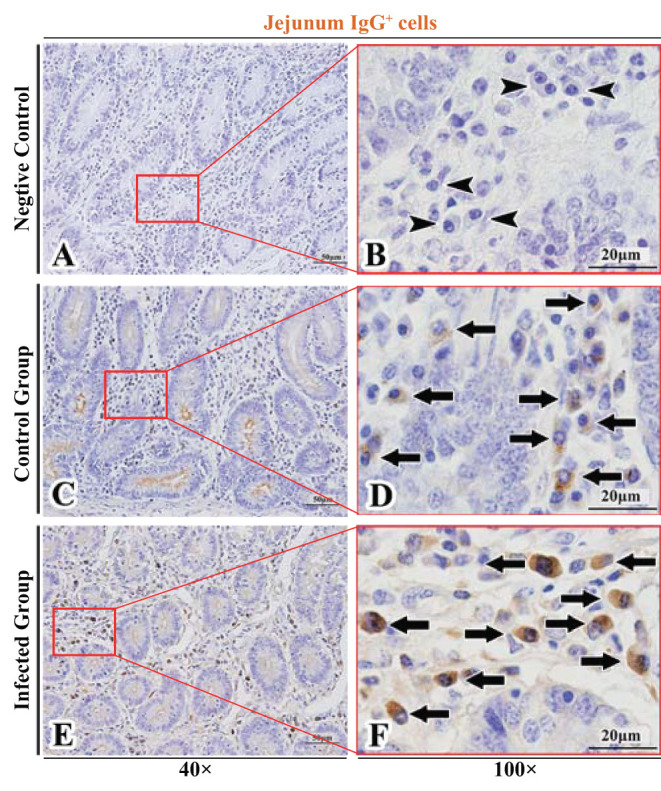
Distribution of IgG^+^ cells in sheep jejunum. **(A,B)** are negative controls; **(C,D)** are Control groups; **(E,F)** are Infected groups. Triangular arrows indicated plasma cells, arrows indicated IgG^+^ cells, and the right image (Scale bar, 20 μm) is a magnification from the part of the left image (Scale bar, 50 μm).

**Figure 7:**
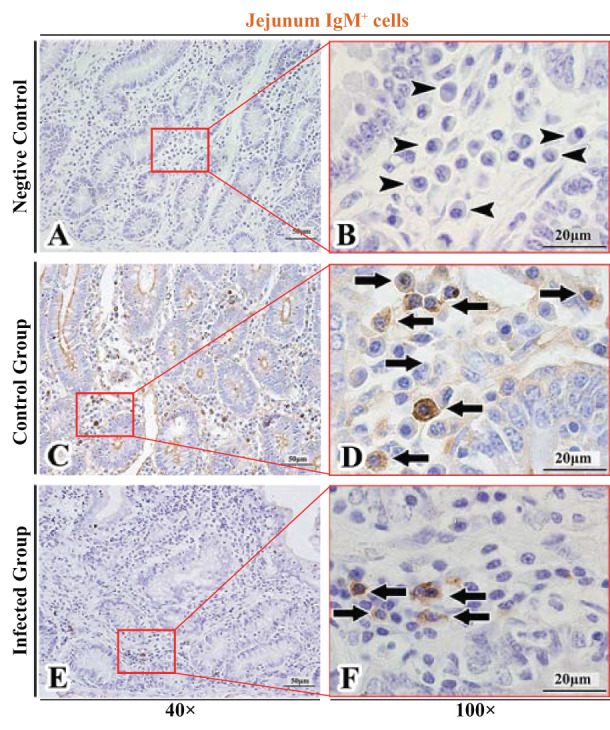
Distribution of IgM^+^ cells in sheep jejunum. **(A,B)** are negative controls; **(C,D)** are Control groups; **(E,F)** are Infected groups. Triangular arrows indicated plasma cells, arrows indicated IgM^+^ cells, and the right image (Scale bar, 20 μm) is a magnification from the part of the left image (Scale bar, 50 μm).

**Figure 8:**
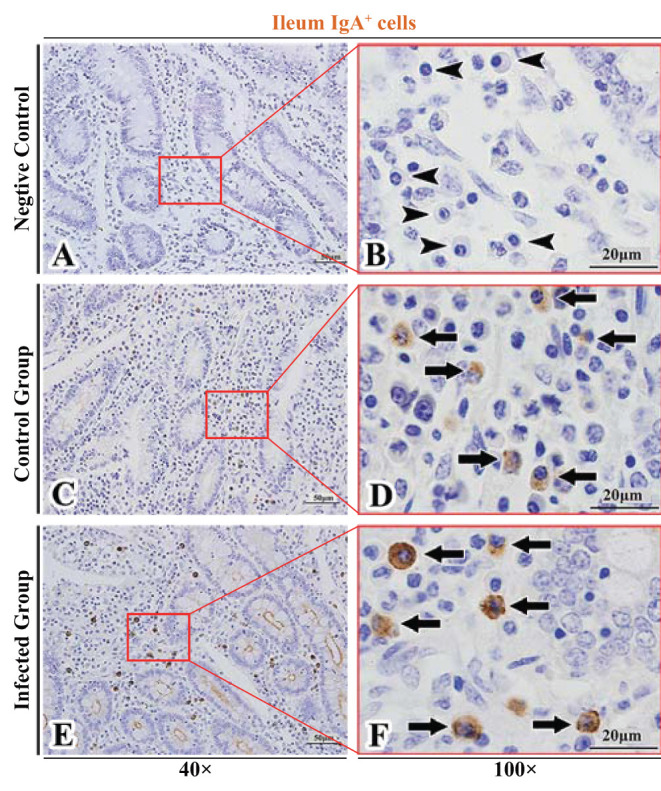
Distribution of IgA^+^ cells in sheep ileum. **(A,B)** are negative controls; **(C,D)** are Control groups; **(E,F)** are Infected groups. Triangular arrows indicated plasma cells, arrows indicated IgA^+^ cells, and the right image (Scale bar, 20 μm) is a magnification from the part of the left image (Scale bar, 50 μm).

**Figure 9:**
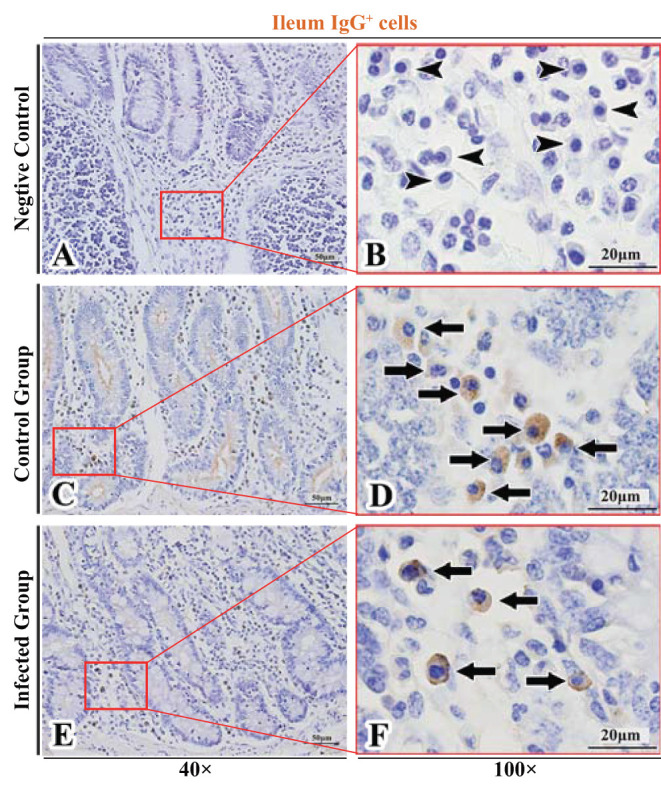
Distribution of IgG^+^ cells in sheep ileum. **(A,B)** are negative controls; **(C,D)** are Control groups; **(E,F)** are Infected groups. Triangular arrows indicated plasma cells, arrows indicated IgG^+^ cells, and the right image (Scale bar, 20 μm) is a magnification from the part of the left image (Scale bar, 50 μm).

**Figure 10:**
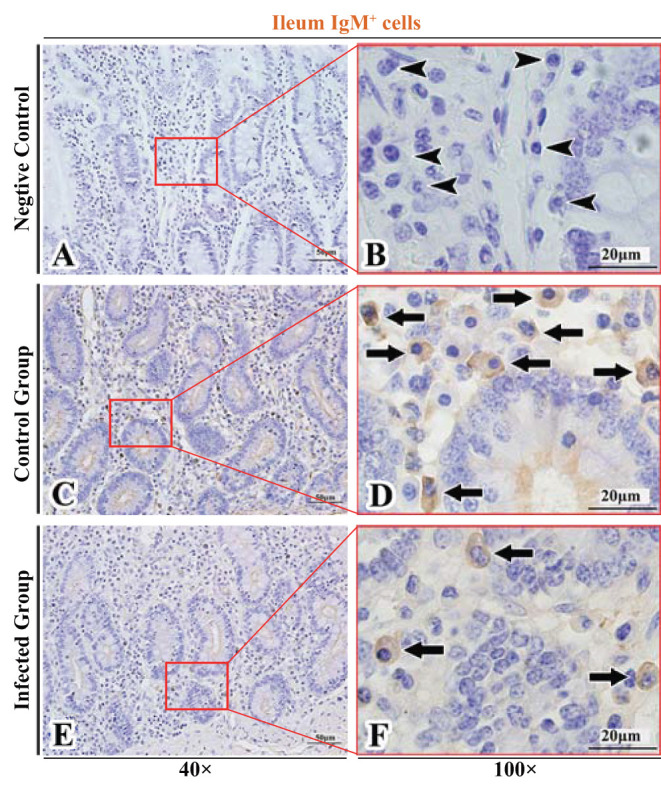
Distribution of IgM^+^ cells in sheep ileum. **(A,B)** are negative controls; **(C,D)** are Control groups; **(E,F)** are Infected groups. Triangular arrows indicated plasma cells, arrows indicated IgM^+^ cells, and the right image (Scale bar, 20 μm) is a magnification from the part of the left image (Scale bar, 50 μm).

**Figure 11:**
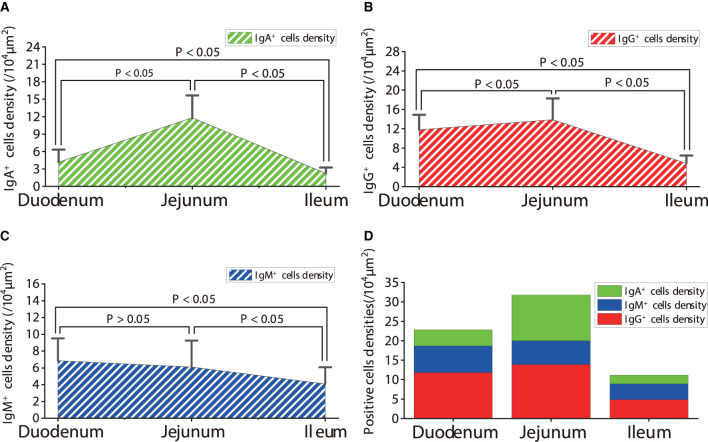
Density of IgA^+^, IgG^+^ and IgM^+^ cells in the small intestine of healthy sheep. **(A)** The distribution density of IgA^+^ cells; **(B)** The distribution density of IgG^+^ cells; **(C)** The distribution density of IgM^+^ cells; **(D)** The total densities of IgA^+^ cells, IgG^+^ cells, and IgM^+^ cells. *P* < 0.05 means significant difference, *P* > 0.05 means not significant difference.

The observation results showed that IgA^+^, IgG^+^, and IgM^+^ cells were mainly distributed in the LP of duodenum (Figures 2A–D, 3A–D, 4A–D), jejunum (Figures 5A–D, 6A–D, 7A–D) and ileum (Figures 8A–D, 9A–D, 10A–D) in sheep and were all distributed diffusely. The distribution densities statistics showed that the densities of IgA^+^ cells in the duodenum, jejunum and ileum were 4.17 cells/10^4^ μm^2^, 11.83 cells/10^4^ μm^2^, and 2.22 cells/10^4^ μm^2^, respectively, and varied greatly among different intestine segments (Figure 11A); the densities of IgG^+^ cells in the duodenum, jejunum and ileum were 11.80/10^4^ μm^2^, 13.87/10^4^ μm^2^, and 4.84/10^4^ μm^2^, and varied greatly among different intestine segments (*P* < 0.05) (Figure 11B); the densities of IgM^+^ cells in the duodenum, jejunum and ileum were 6.85 cells/10^4^ μm^2^, 6.09 cells/10^4^ μm^2^, and 4.08 cells/10^4^ μm^2^, respectively, there were not significant difference between duodenum and jejunum (*P* > 0.05), but they were significantly higher than those in ileum (*P* < 0.05) (Figure 11C). The total distribution density of IgA^+^, IgG^+^, and IgM^+^ cells in the jejunum was highest, followed by the duodenum and ileum (Figure 11D).

### The Distribution of IgA^+^, IgG^+^, and IgM^+^ Cells in the Small Intestine of Sheep After Infection With *Moniezia benedeni*

**Figure 12:**
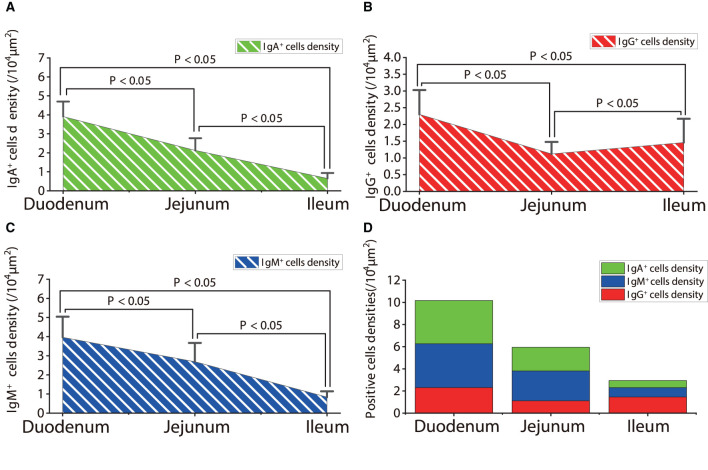
The distribution densities of IgA^+^, IgG^+^, and IgM^+^ cells in the small intestine of sheep after infection with *Moniezia benedeni*. **(A)** The distribution density of IgA^+^ cells; **(B)** The distribution density of IgG^+^ cells; **(C)** The distribution density of IgM^+^ cells; **(D)** The total densities of IgA^+^, IgG^+^, and IgM^+^ cells. *P* < 0.05 means significant difference.

The observation results showed that in the small intestine of sheep infected with *Moniezia benedeni*, IgA^+^, IgG^+^, and IgM^+^ cells were also all mainly diffusely distributed in the LP of the duodenum (Figures 2E,F, 3E,F, 4E,F), the jejunum (Figures 5E,F, 6E,F, 7E,F) and ileum (Figures 8E,F, 9E,F, 10E,F). Distribution densities statistics showed that the densities of IgA^+^ cells were 2.30 cells/10^4^ μm^2^, 1.12 cells/10^4^ μm^2^, and 1.46 cells/10^4^ μm^2^, respectively, and the differences among them were significant (*P* < 0.05) (Figure 12A); the densities of IgG^+^ cells were 3.96 Cells/10^4^ μm^2^, 2.69 cells/10^4^ μm^2^, 0.84 cells/10^4^ μm^2^, and the differences among them were also significant (*P* < 0.05) (Figure 12B); the densities of IgM^+^ cells were 3.91 cells/10^4^ μm^2^, 2.13 cells/10^4^ μm^2^, and 0.65 cells/10^4^ μm^2^, respectively, and the differences among them were significant (*P* < 0.05) (Figure 12C). The total distribution densities of IgA^+^, IgG^+^, and IgM^+^ cells in the duodenum, jejunum and ileum were gradually decreased in turn (Figure 12D).

### Influence of *Moniezia benedeni* Infection on the Distribution of IgA^+^, IgG^+^, and IgM^+^ Cells

**Figure 13:**
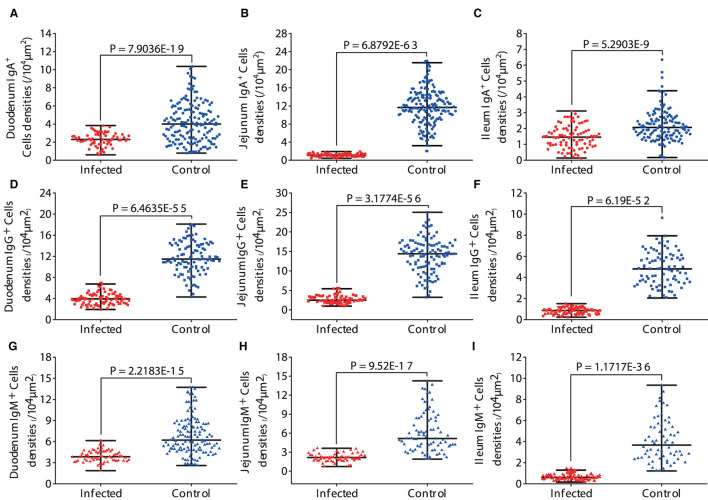
The impact of *Moniezia benedeni* infection on the distribution of IgA^+^, IgG^+^, and IgM^+^ cells in the small intestine of sheep. **(A–C)** The impact of *Moniezia benedeni* infection on IgA^+^ cells density; **(D–F)** The impact of *Moniezia benedeni* infection on IgG^+^ cells density; **(G–I)** The impact of *Moniezia benedeni* infection on IgM^+^ cells density. *P* < 0.05 means significant difference.

The observation results showed that *Moniezia benedeni* infection did not change the characteristics of the diffuse distribution of IgA^+^, IgG^+^, and IgM^+^ cells in the LP of the sheep small intestine (Figures 2–10). The distribution densities statistics showed that after *Moniezia benedeni* infection, the total densities in the three intestine segments were all dropped significantly (Figure 13). In duodenum, jejunum and ileum, IgA^+^ cells were decreased by 44.84%, 90.53%, and 34.23%, respectively; IgG^+^ cells were decreased by 66.44%, 80.61%, and 82.64%, respectively; and IgM^+^ cells were reduced by 42.92%, 65.02%, and 84.07%, respectively (Table 1).

**Table 1:** Decrease rate of IgA^+^, IgG^+^, and IgM^+^ cells in the small intestine of sheep infected with *Moniezia benedeni* (%).

	**Duodenum**	**Jejunum**	**Ileum**
IgA^+^ cells density	44.84%	90.53%	34.23%
IgG^+^ cells density	66.44%	80.61%	82.64%
IgM^+^ cells density	42.92%	65.02%	84.07%

Decrease rate = (A-B)/A^*^100%. (A Positive cells density in Control group; B Positive cells density in infected group).

## Discussion

IgA^+^, IgG^+^, and IgM^+^ cells are important members of gastrointestinal mucosal immunity. The results of the present study showed that the distribution of IgA^+^ cells and IgG^+^ cells were significantly different (*P* < 0.05) among the duodenum, jejunum, and ileum, except for not significant difference in IgM^+^ cells was between the duodenum and jejunum (*P* > 0.05). From the duodenum to the ileum, on the one hand, due to the differences in the composition of intestinal tissue, such as the numbers of intestinal absorption epithelium, goblet cells, Paneth cells, tuft cells, and endocrine cells in different intestinal segments (51); on the other hand, due to the differences in intestinal environment, such as diet, oxygen levels, bile acids, transit time of chyme, pH, and thickness of the covering mucus (52), the small intestine is endowed with regional specificity. This difference has a considerable impact on the abundance, diversity and function of the microorganisms that colonizing the intestine. For instance, the colonized microbial community is about 10^1^~10^3^ CFU/ml in the duodenum (53) and 10^4^~10^7^ CFU/ml in the jejunum (mainly including bacteria represented by Firmicutes, also including Proteobacteria, Actinomycetes, and Bacteroidetes) (53, 54), 10^3^~10^8^ CFU/ml in the ileum (mainly composed of Bacteroidetes, *Clostridium*, Enterobacteriaceae, *Lactobacillus* and *Veillonella*) (55, 56). Further studies have shown that this differential gut microbiota also has an important impact on the function of the gut. For instance, fecal microbial transplantation in the jejunum of germ-free mice could cause functional changes in the jejunum transcriptome. Under closed conditions, specific microbial community members could alter the genetic profile of the ileum (54). More importantly, the gut microbiota has an important impact on host immune function, such as B. *thetaiotamicron* could significantly increase the colipase gene expression in ileal crypts and many other genes expression related with intestinal barrier functions and immune responses (57). Meanwhile, the SIgA and its transported receptor pIgR were both increased after bacterial colonization (57). These reports suggested that innate and adaptive immune functions in the ileum were both significantly affected by the gut microbiome (58). However, some researches have shown that the lymph nodes located in the different intestinal segments perform “segmental” immune regulation on the intestine. For instance, the immune responses were different in the different intestinal segments of mice attacked by pathogens, such as *Salmonella* (37). Therefore, the results of the present study suggested that, under physiological conditions, the distribution of the main antibody-secreting cells that form the immune barrier in the small intestine of sheep presented significant regional specificity, and this specificity was adapted to the changes of their colonized flora. As other reasons for this phenomenon need to be further studied.

Parasites could actively regulate each phase of the host's immune response by releasing various immunomodulatory molecules. The results of this study showed that the three types of cells in each intestinal segment were all dramatically dropped after *Moniezia benedeni* infection. Many studies have confirmed that parasites [such as *Heligmosomoides polygyrus* (59), *S. mansoni; S. haematobium* (60, 61), *F. hepatica* (62–64) etc.] can release a series of finely tuned and highly evolved immune regulators during their parasitic process, mainly including cytokine and innate defenses homologs and growth factors, Toll like receptor (TLR) signaling, intracellular signaling and gene expression, enzymes and inhibitors, lipid or lipid-binding, and extracellular vesicles. They could individually target different phases of immune responses, such as (i) initiating alarmin signals, (ii) antigen recognition and processing, (iii) adaptive responses, (iv) effector cell responses, and (v) coagulation, healing, and remodeling to actively intervene on host immune function (14). *Moniezia benedeni* mainly parasitizes in the small intestine of the host, and its body length could reach more than 4 m (47). Cestodes feed transcuticularly, through osmocytosis processes, the body cuticle being metabolically active (47), the worms could take in large amounts of nutrients from the host intestine, meanwhile, which could secrete large amounts of toxic substances during development (65, 66). Therefore, the metabolites secreted by the worm could also be widely dispersed into the small intestine of the host and inhibit the residence of the IgA^+^, IgG^+^, and IgM^+^ cells in the small intestine of the host. It would facilitate *Moniezia benedeni* to resist or evade the recognition, killing and clearance by the host immune system. As for the different inhibition ratios in different intestinal segments, it may be related to the differences in the types and contents of immunomodulatory products secreted by different parts of *Moniezia benedeni*, including the scolex, neck, and strobila, and which need to be further studied and analyzed.

## Conclusions

SIgA, IgG, and IgM play key roles in the formation of the intestinal mucosal immune barrier. The results suggested that in the healthy sheep intestine, the three types of positive cells were resided heterogeneously in the small intestinal mucosa, exhibiting a significant region-specificity; *Moniezia benedeni* infection could not change the diffuse distribution of them, but could significantly reduce their densities, and the forming mucosal immune barrier were significantly inhibited. This provided powerful evidence for further studying on the mechanism of *Moniezia benedeni* evasion from immune surveillance.

## Data Availability Statement

The original contributions presented in the study are included in the article/supplementary material, further inquiries can be directed to the corresponding author/s.

## Ethics Statement

In this study, all experimental procedures were approved by the Animal Care and Use Committee (IACUC) of College of Veterinary Medicine of Gansu Agricultural University (approval no.: GSAU-Eth-VMC-2021-021). All efforts were made to minimize suffering.

## Author Contributions

W-LY and W-DZ proposed the study framework, designed, developed the experiment, and analyzed the results. L-XH, JP, and B-SW participated in the experiments. W-HH, X-PF, and W-HW monitored the progress of the study. All authors read and approved the manuscript.

## Funding

This study was supported by grant sponsor: National Natural Science Foundation of China; grant number: 31902235. Special funds for discipline construction; grant number: GAU-XKJS-2018-10. Scientific research start-up funds for openly-recuited doctors; grant number: 2017RCZX-12. Projects to improve the innovation ability of colleges and universities in Gansu Province; grant number: 2019B-070.

## Conflict of Interest

The authors declare that the research was conducted in the absence of any commercial or financial relationships that could be construed as a potential conflict of interest.

## Publisher's Note

All claims expressed in this article are solely those of the authors and do not necessarily represent those of their affiliated organizations, or those of the publisher, the editors and the reviewers. Any product that may be evaluated in this article, or claim that may be made by its manufacturer, is not guaranteed or endorsed by the publisher.

## Abbreviations

IgA: Immunoglobulin A
SIgA: secreted immunoglobulin A
pIgR: Poly immunoglobulin receptor
FcRn: neonatal Fc receptor
ILC2s: group 2 innate lymphoid cells
LP: *lamina propria*
TLR: Toll like receptor.
